# Mapping of the transcription start site (TSS) and identification of SNPs in the bovine neuropeptide Y (*NPY*) gene

**DOI:** 10.1186/1471-2156-9-91

**Published:** 2008-12-23

**Authors:** Bojlul Bahar, Torres Sweeney

**Affiliations:** 1 Cell and Molecular Biology Lab, School of Agriculture, Food Science & Veterinary Medicine, Veterinary Science Centre, University College Dublin, Belfield, Dublin 4, Ireland

## Abstract

**Background:**

Neuropeptide Y is a key neurotransmitter of the central nervous system which plays a vital role in the feed energy homeostasis in mammals. Mutations in the regulatory and coding regions of the bovine *NPY *gene can potentially affect the neuronal regulation of appetite and feeding behaviour in cattle. The objectives of this experiment were to: a) fully characterize the bovine *NPY *gene transcript and b) identify the SNP diversity in both coding and non-coding regions of the *NPY *gene in a panel of *Bos taurus *and *B. indicus *cattle.

**Results:**

Bovine *NPY *gene consists of four exons (99, 188, 81 and 195 nucleotides) and three introns. The promoter region of the *NPY *gene consists of TATA and GC boxes which are separated from the transcription start site (TSS) by 29 and ~100 nt, respectively. Analyses of the tissue specific expression of the bovine *NPY *gene revealed the presence of highly abundant *NPY *gene transcripts in the arcuate nucleus, cerebral and cerebellar regions of the bovine brain. We identified a total of 59 SNPs in the 8.4 kb region of the bovine *NPY *gene. Seven out of nine total SNPs in the promoter region affect binding of putative transcription factors. A high level of nucleotide diversity was evident in the promoter regions (2.84 × 10^-3^) compared to the exonic (1.44 × 10^-3^), intronic (1.30 × 10^-3^) and 3' untranslated (1.26 × 10^-3^) regions.

**Conclusion:**

The SNPs identified in different regions of bovine *NPY *gene may serve as a basis for understanding the regulation of the expression of the bovine *NPY *gene under a variety of physiological conditions and identification of genotypes with high feed energy conversion efficiency.

## Background

Following concerns that methane emissions from ruminants are contributing to global warming, there is an increased demand for the development of sustainable agricultural production systems [1,2]. One component of such systems is the ability to be able to genetically select animals that can efficiently utilize feed energy [3,4]. This requires an understanding of the underlying genomic factors influencing energy conversion efficiency in ruminants. Neuropeptide Y, a key neurotransmitter of the central nervous system which plays a vital role in the feed energy homeostasis in mammals, has the potential to serve as a candidate for the energy conversion in ruminants. Expression of the neuropeptide Y (*NPY*) gene is considered to be a regulator of feed intake in cattle [5]. Neuropeptide Y influences a number of biological pathways regulating appetite, feeding behavior and energy homoeostasis in humans and animals [6-9]. Neuropeptide Y was found to stimulate growth hormone secretion in cattle [10], to increase the expression of the leptin gene [11] in sheep, and to decrease free fatty acid secretion in adipocyte cell lines [12].

In cattle, the *NPY *gene is mapped to chromosome 4 [13]. The *NPY *gene codes for a functional peptide of 36 amino acid residues. This peptide is highly conserved across the mammalian species further indicating the physiological importance of the neuropeptide Y molecule [14]. It is likely that because of evolutionary conservation, the *NPY *gene harbors useful genetic variation in the regulatory regions [15]. To identify the promoter region and to distinguish between the coding and non-coding regions of the gene, it is important that the transcription start site (TSS) be accurately identified [16]. *In silico *identification of the TSS through computational methods is feasible, but such methods need further experimental verification [17]. A comparative genomic analysis, combined with the sequence information derived from the full length cDNA is identified as a technique of identification of the TSS [18-20].

Three SNPs were previously identified in the intronic regions of the bovine *NPY *gene [13]. However, currently no information is available on the extent of genetic diversity present in the promoter and exonic regions of the bovine *NPY *gene. SNPs in the promoter region may affect binding of transcription factors and thus influence the expression of the *NPY *gene. Hence, the objectives of this experiment were to: a) fully characterize the bovine *NPY *gene transcript and b) identify the SNP diversity in both coding and non-coding regions of the *NPY *gene in a panel of *Bos taurus *and *B. indicus *cattle.

## Results

### *In silico *comparative sequence analysis of *NPY *gene

The NCBI/Ensemble available 5' untranslated region and the first available exon of the bovine *NPY *gene [GeneBank: AY491054] were compared to the orthologous sequences from the horse [Ensembl: ENSECAG00000008726], mouse [Ensembl: ENSMUSG00000029819], rat [Ensembl: ENSRNOG00000009768], dog [Ensembl: ENSCAFG00000002801] and human [Ensemble: ENSG00000122585]. Comparative sequence analysis revealed highly conserved sequences among the mammalian species with 100% homology in a GC rich (GGGGCGGG) region and an AT rich (ATAAAA) region. These two regions most likely represent the GC box and TATA box components of the core promoter of the mammalian *NPY *gene.

However, a discrepancy was identified in the naming of the exons. Exon 1 of the horse, mouse and human, corresponded to a section of the 5' untranslated region (UTR) in the bovine sequence. The first exon reported in the NCBI bovine sequence was 1056 nucleotides (nt) downstream from the TATA box. As the core promoter sequence (TATA box) of a typical TATA driven mammalian gene is generally located about 26 to 31 nt upstream of the TSS [21], we sought to verify the presence of an additional currently unidentified exon containing the TSS in the bovine.

### Identification of exon 1 and the TSS of the bovine *NPY *gene

**Figure 1:**
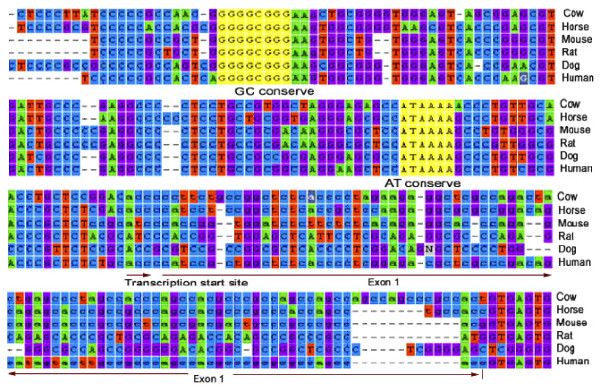
**Comparative sequence analyses of the promoter region of the *NPY *gene in mammalian species**. The conserved TATA and GC boxes (highlighted in yellow), transcription start sites and the available exon1 (in lower case) are shown.

**Figure 2:**
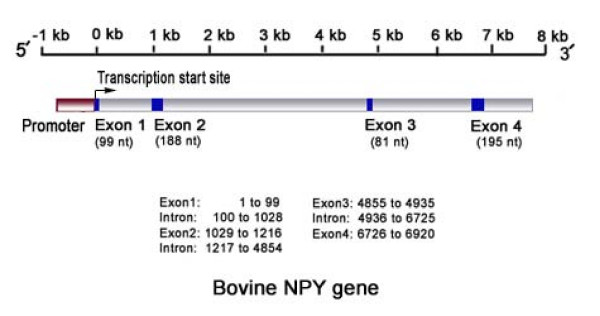
**Schematic diagram of the bovine *NPY *gene showing the promoter, exons, introns and 3' regions and their relative sizes and positions reference to the TSS**.

Analysis of the sequence data from full length cDNA [GeneBank: EU935419] from the arcuate nucleus, cerebral, and cerebellar regions of the brain revealed that the TSS of the bovine *NPY *gene (comprised of an ACC site) was located 29 nt downstream of the ATAAAA motif (relative to the T). Identification of the TSS confirms that a previously unidentified exon is present in the bovine *NPY *gene. Comparative sequence analysis of full length cDNA with the NCBI available DNA sequences from the horse, mouse, rat, dog and human identified this exon (99 nt length) as exon 1 in the bovine (Figure 1). This confirms that the bovine *NPY *gene consists of four exons (total cDNA length 563 nt) and three introns (Figure 2).

### Variation in the expression of *NPY *gene in different tissues

**Figure 3:**
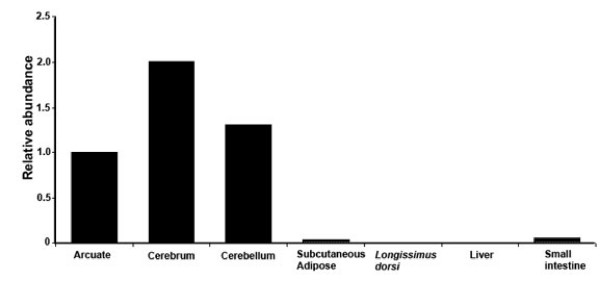
**Relative abundance of bovine *NPY *gene transcript in the arcuate, cerebrum and cerebellum regions of the brain, subcutaneous adipose tissue, *Longissimus dorsi*, liver and small intestine tissues**. The mRNA transcripts of bovine *NPY *and *GAPDH *genes were quantified by the real time reverse transcriptase PCR. The relative quantities of the mRNA transcript of bovine *NPY *gene were normalized against the relative quantities of the *GAPDH *(housekeeping) gene.

The expression of the bovine *NPY *gene was investigated in the arcuate nucleus, cerebral and cerebellar regions of the brain, subcutaneous adipose tissue, *Longissimus dorsi *muscle, liver and small intestine. As demonstrated by the qualitative gene expression analysis by RT-PCR, the mRNA of the bovine *NPY *gene was present in the arcuate nucleus, cerebral and cerebellar regions of the brain, subcutaneous adipose tissue, *L. dorsi *muscle and small intestine and absent in the liver (data not shown). However, the quantitative real time RT-PCR revealed that the relative abundance of the *NPY *gene transcript (normalized against the *GAPDH *gene), were high in the brain tissues, low in the subcutaneous adipose tissue and small intestine and negligible in the *L. dorsi *muscle (Figure 3).

### SNPs in the promoter region of the bovine *NPY *gene

**Figure 4:**
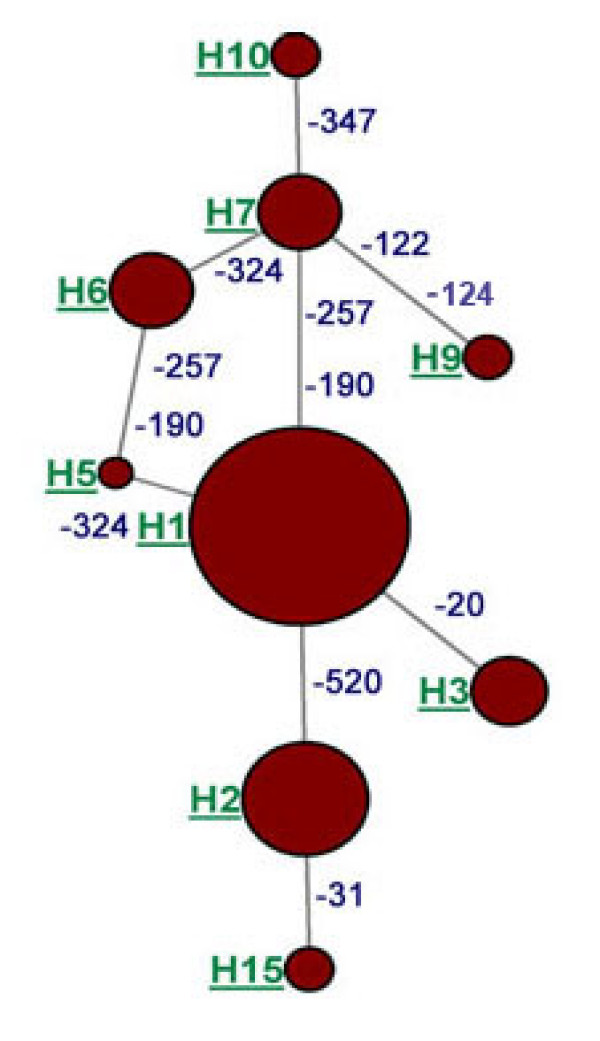
**Diversity of the haplotypes based on the occurrence of SNPs in the promoter region of the *NPY *gene in the *Bos taurus *and *B. indicus *cattle**. The major haplotypes (H1, H2, H3, H5, H6, H7, H9, H10 and H15) are indicated by the circle proportional to the haplotype frequency and separated by the SNP loci differing between the haplotypes.

The SNPs identified were positioned based on the identification of the TSS. Nine SNPs were identified in a 639 nt segment of the promoter region of the bovine *NPY *gene (Table 1). The nucleotide diversity in this region was calculated to be 2.84 × 10^-3^. Of these SNPs, four (-20, -122, -124 and -347) were unique to *B. taurus *and one (-31) was unique to *B. indicus*. Four SNPs (-190, -257, -324 and -520) were common to both species. At loci -257 and -190, A and T were the major alleles in *B. taurus *whereas in these two loci C was the major allele in *B. indicus*. Two C insertions, at -122 and -124 were present in one Limousin animal. Using EM algorithm a total of 15 haplotypes were identified from the pooled datasets from *B. taurus *and *B. indicus *samples and the haplotypes diversity presented (Figure 4).

**Table 1:** Single nucleotide polymorphisms (SNPs) identified in the promoter region of bovine *NPY *gene and their population statistics.

NCBI dbSNP Accession ss#	Locus	SNP	*Bos taurus (n = 34)*		*Bos indicus (n = 6)*	
		
			Major allele frequency	Heterozygous	Major allele frequency	Heterozygous
107795824	-520	C/A	0.85 (C)	9	0.50 (C)	0
107795825	-347	A/T	0.97 (A)	0	-	-
107795826	-324	C/A	0.97 (C)	2	0.50 (C)	1
107795827	-257	A/C	0.95 (A)	1	0.90 (C)	1
107795828	-190	T/C	0.95 (T)	1	0.90 (C)	1
107795829	-124	C/+	0.97 (C)	0	-	-
107795830	-122	C/+	0.97 (C)	0	-	-
107795831	-31	C/+	-	-	0.83 (C)	0
107795832	-20	T/G	0.93 (T)	1	-	-

The promoter sequence of the bovine *NPY *gene was screened for the identification of different putative transcription factor binding sites (TFBS). In the 639 nt promoter region examined, a total of 61 TFBS bindings were predicted (core similarity >80% and matrix similarity >85%, both strands) which included TFBS from 51 different transcription factor families (data not shown). SNPs at loci -20, -31, -122, -124, -257, -324 and -347 affect 15 putative TFBS (Table 2). Of the total 9 SNPs identified in the promoter region of the bovine *NPY *gene, the SNPs at -20 and -31 are very interesting since they are present in the core promoter region (TATA box). These two SNPs are most likely to affect the binding of the TATA-binding protein factor and autoimmune regulatory element binding factor.

**Table 2:** The SNPs in the 639 nt promoter region of bovine *NPY *gene those affects the putative transcription factors

**Putative transcription factors**	**Start**	**End**	**Recognition sequence**	**SNPs**
PAX-4/PAX-6 paired domain binding sites	-356	-338	gagatagg**t**GCAGcccaga*	A/T (-347)
GATA binding factors	-348	-336	cagaGATAgg**t**gc*	A/T (-347)
Nuclear factor 1	-341	-321	cc**g**gtctagtcctGCCAgaga*	C/A (-324)
Vertebrate SMAD family of transcription factors	-331	-323	**g**GTCTagtc*	C/A (-324)
E-box binding factors	-324	-312	cctCCACgccc**g**g*	C/A (-324)
Nuclear receptor subfamily 2 factors	-280	-256	**t**ggaccacgcCAAAgcacaagtttc*	A/C (-257)
EGR/nerve growth factor induced protein C & related factors	-135	-119	aagg**g**A**G**GGggtccaga*	+C (-122), +C(-124)
EGR/nerve growth factor induced protein C & related factors	-133	-117	ataaGG**G**A**g**ggggtcca*	+C (-122), +C(-124)
Pleomorphic adenoma gene	-132	-112	GGGGgataagg**g**a**g**ggggtcc*	+C (-122), +C(-124)
Myc associated zinc fingers	-131	-119	aagg**g**a**G**GGGgtc*	+C (-122), +C(-124)
GC-Box factors SP1/GC	-131	-117	ataagG**G**A**G**ggggtc*	+C (-122), +C(-124)
Gut-enriched Krueppel like binding factor	-128	-116	gataagg**g**a**G**GGG*	+C (-122), +C(-124)
GATA binding factors	-124	-112	ggggGATAagg**g**a*	+C (-124)
Autoimmune regulatory element binding factor	-38	-12	ttgcaac**a**gggtttttaT**G**GCtctccc*	+C (-31), T/G(-20)
Tata-binding protein factor	-33	-17	agc**c**aTAAAaaccc**t**gt	+C (-31), T/G(-20)

*- minus strand

### SNPs in the exonic, intronic and 3' UTR of the bovine *NPY *gene

In the 563 nt exonic region (total 4 exons) of the bovine *NPY *gene, a total of four SNPs were identified (Table 3). The nucleotide diversity in the exonic regions was calculated to be 1.44 × 10^-3^. The SNPs at +1100 (*B. taurus*) and +1106 (*B. indicus*) were present in exon 2. However, as deduced from the amino acid sequence of the *NPY *peptide, no amino acid changes occur due to any of these SNPs. Two exonic SNPs were also present at +6751 (*B. taurus*) and +6887 (*B. indicus*) in exon 4. These variants are located in the untranslated region of the mRNA transcript which might have regulatory role.

**Table 3:** Single nucleotide polymorphisms (SNPs) in the exonic (in bold letters), intronic and 3' regions of bovine neuropeptide Y (NPY) gene.

NCBI dbSNP Accession ss#	Locus	SNP	*Bos taurus *(n = 34)		*B. indicus *(n = 6)	
		
			Major allele frequency	Heterozygous	Major allele frequency	Heterozygous
107795833	197	C/G	-	-	0.83	0
107795834	218	G/C	0.91	0	0.83	0
107795835	228	C/T	0.99	1	0.67	4
107795836	305	C/G	0.93	3	0.92	1
107795837	358	G/A	0.99	1	0.75	3
107795838	361	A/C	0.93	3	0.92	1
107795839	380	G/A	-	-	0.67	4
107795840	592	C/A	0.99	1	0.67	2
107795841	642	G/A	0.93	3	0.88	1
107795842	762	T/C	0.96	1	0.92	1
107795843	820	G/A	0.97	0	-	-
107795844	1004	C/+	-	-	0.83	0
107795845	1014	C/T	0.97	2	0.83	0
107795846	**1100**	**C/T**	**0.99**	**1**	**-**	**-**
107795847	**1106**	**C/G**	**-**	**-**	**0.90**	**1**
107795848	1312	A/C	0.75	9	-	-
107795849	1321	A/G	0.87	5	-	-
107795850	1420	C/A	-	-	0.90	1
107795851	1541	G/A	-	-	0.80	2
107795852	1694	A/G	0.57	11	-	-
107795853	1713	G/T	0.85	4	-	-
107795854	1757	G/A	-	-	0.80	2
107795855	1865	T/C	0.93	3	-	-
107795856	2175	C/T	0.91	4	-	
107795857	2551	A/-	-	-	-	-
107795858	2892	G/A	0.70	14	-	-
107795859	3883	A/-	-	-	-	-
107795860	4018	G/A	0.97	0	-	-
107795861	4042	G/A	-	-	0.80	2
107795862	4066	T/C	0.75	9	-	-
107795863	4953	G/A	0.96	3	-	-
107795864	5213	C/G	0.96	3	-	-
107795865	5273	A/G	0.96	3	-	-
107795866	5280	T/A	0.96	3	-	-
107795867	5642	C/T	0.95	3	0.75	1
107795868	5930	A/G	-	-	0.83	2
107795869	6341	G/A	-	-	0.50	4
107795870	6368	T/G	0.95	3	0.75	1
107795871	6385	A/G	0.95	3	0.75	1
107795872	6400	C/T	0.95	3	0.75	1
107795873	6526	A/T	0.98	1	-	-
107795874	6527	G/A	0.95	3	-	-
107795875	6658	A/C	0.91	4	0.83	2
107795876	**6751**	**T/G**	**0.99**	**1**	**-**	
107795877	**6887**	**A/T**	**-**	**-**	**0.83**	**2**
107795878	7192	C/T	0.50	16	-	-
107795879	7211	A/T	0.91	4	-	-
107795880	7552	T/-	0.88	0	0.80	0
107795881	7553	C/-	0.88	0	0.80	0
107795882	7641	T/G	0.88	2	-	-

A total of forty one SNPs were identified in the 6358 nt region across the three introns (Table 3). Thirteen SNPs were identified in intron 1 (929 nt), fifteen in intron 2 (3638 nt) and thirteen in intron 3 (1790 nt). The overall nucleotide diversity in the intronic regions was 1.30 × 10^-3^. A total of five SNPs were also identified in the 3' UTR (801 nt) of the bovine *NPY *gene (Table 3) which gave rise to a nucleotide diversity value of 1.26 × 10^-3^.

## Discussion

Following a combined approach of full length cDNA analyses, genomic DNA sequencing and evolutionary genomics, we characterised the bovine *NPY *gene transcript. We identified one previously unidentified exon of the bovine *NPY *gene and mapped the TSS and various coding and non-coding regions of the *NPY *gene transcript. Analyses of the tissue specific expression of the bovine *NPY *gene revealed the presence of highly abundant *NPY *gene transcripts in the arcuate nucleus, cerebral and cerebellar regions of the bovine brain. We sequenced a total of 8361 nt genomic region which included the coding and non-coding regions of the gene and identified a total of 59 SNPs. A high level of nucleotide diversity was evident in the promoter region compared to the exonic, intronic and 3' UTR.

Based on the *in silico *analyses of the *NPY *gene sequences from horse, mouse, rat, dog and human, we identified the core promoter of the bovine *NPY *gene. Our results suggested that the conserved ATAAAA region of the *NPY *gene is the TATA element. The TATA element serves as a binding site for the TATA binding protein (TBP), an important transcription factor which plays a key role in the transcriptional activity of the RNA polymerase II enzyme [21,22]. We also identified another interesting conserved GC rich region (GGGGCGGG) as the GC box element of the *NPY *promoter. The mammalian GC box element is the TFBS of SP1 which initiates transcription [23-25]. The TATA and the GC elements are separated from the TSS by 29 nt and ~100 nt, respectively, which is typical for any TATA containing mammalian gene [21]. The previously reported TATA elements of the *NPY *gene of chicken (TTAAAA) [14] and Chinese perch (ATAAAA) [26] were very similar to the TATA element of the mammals identified in this experiment. The TSS of the bovine *NPY *gene (ACC) is similar to the previously characterised TSS of the human [27].

We identified an extra exon of the bovine *NPY *gene (99 nt length) which was previously uncharacterized. In the NCBI/Ensemble databases, this exon was designated in the horse, mouse and human *NPY *gene sequences. This exon is located outside the region that translates into the core peptide of 36 amino acids. As in most eukaryotic genes, the region corresponding to the first exon in the mRNA transcript is likely to play a regulatory role in the translation of the *NPY *mRNA [28]. The first exon provides the initial binding site for the 40S ribosomal unit which is required to start the translation at an AUG codon of the reading frame [28]. To have an efficient translation at the AUG codon, the first exon must have an A at three nt upstream of the AUG codon [28,29]. This A was highly conserved across the mammalian species investigated in this experiment. The length of the bovine *NPY *cDNA deduced (563 nt, excluding the poly A tail) is in agreement with the previously reported *NPY *cDNA length of the human (591 nt, which included the poly A tail) [27].

A high level of expression of the *NPY *gene was evident in the arcuate nucleus, cerebral and cerebellar regions of the brain. As previously demonstrated in the mammalian brain, Neuropeptide Y is widely distributed in the central nervous system of mammals [30,31]. The availability of the *NPY *mRNA transcript in the central nervous system suggests its central regulation of energy homeostasis. Among the peripheral tissues, expression of the *NPY *gene was also evident in the subcutaneous adipose tissue and small intestine. Since both subcutaneous adipose tissue and the mammalian gastrointestinal tract are innervated, such tissues can also produce neuropeptide Y [26,32]. Recently, mammalian adipose tissue has been identified as a new site of *NPY *gene expression and neuropeptide Y biosynthesis [33,34]. The neuropeptide Y produced in the brain and that of the peripheral tissue system such as adipose tissues had structural similarity. However, the functional relevance of neuropeptide Y in these tissues needs more research. Although we did not detect any *NPY *gene transcript in the liver tissue and a minor expression the *L. dorsi*, availability of neuropeptide Y in the mammalian skeletal muscle tissue and liver has been previously reported in the literature [35-37].

We sequenced a 639 nt promoter region and identified nine novel SNPs of which seven SNPs were affecting TFBS. Interestingly, the SNPs at -20 (T to G transversion) and -31 (C addition) are on the TFBS of the TATA binding protein (TBP) transcription factor. The TATA box of the *NPY *gene constitutes the core promoter region which is highly conserved across the mammalian species and hence, the SNPs at -20 and -31 are likely to play vital roles in the expression of the bovine *NPY *gene. Other mutations identified in the promoter region of the bovine *NPY *gene are also likely to affect the expression of the gene and consequently the amount of Neuropeptide Y synthesised. It has potential consequences for feed energy conversion and energy homeostasis in the bovine [38,39]. The haplotypes derived from the SNPs in the promoter region of the bovine *NPY *gene will serve as a basis for developing *in vitro *assays for investigating the role of the promoter SNPs in the regulation of the expression of the bovine *NPY *gene.

Among the four exonic SNPs reported, two exonic SNPs are synonymous and the other two SNPs located in the untranslated region might have a mostly regulatory function which needs further investigation. Among the total 59 SNPs identified in the coding and non-coding regions of the bovine *NPY *gene, three intronic SNPs (+1312, +1694 and +4066) were previously reported [13] and the genetic association of two of these SNPs were recently investigated in beef cattle [5].

Noticeably high nucleotide diversity in the promoter region compared to the exonic, intronic and 3' (UTR) regions of the bovine *NPY *gene observed could serve as a source of genetic variation necessary for the evolutionary adaptation of animals [40]. Since neuropeptide Y is highly conserved during the evolutionary processes, SNPs causing any alteration of the core peptide is probably undesirable [15]. Therefore, the promoter region of the gene is likely to contain more genetic variation for evolutionary adaptation compared to the other regions [15,41]. Although most of the SNPs identified were in the intronic regions, the overall nucleotide diversity in the introns was less than that of the promoter region.

## Conclusion

Characterization of the gene transcript, especially identification of the correct promoter region (*cis *acting element) and TSS, is a requisite for understanding the regulation of gene expression and underlying effects of genetic variation [16]. We systematically characterized the bovine *NPY *gene transcripts and mapped the promoter, TSS and the exons and intron regions. This will serve as a basis for investigating the regulatory role of the SNPs in the expression of the bovine *NPY *gene and identification of genotypes with high feed energy conversion efficiency. The extra exon reported may likely play an important role as a regulatory sequence of gene expression [16]. The SNPs and the haplotypes identified in the bovine *NPY *gene could be targeted for further functional verification and investigating their association with feed intake in cattle in particular, and understanding the physiological role played by the neuropeptide Y in energy homeostasis in mammals, in general.

## Methods

### Tissue samples and RNA extraction

Tissue samples (2 g each) from bovine brain (arcuate nucleus, cerebral and cerebellar regions), subcutaneous adipose tissue, *Longissimus dorsi *muscle, liver and small intestine were surgically removed and collected in RNA *later*™ (Ambion, Inc. Austin, TX). Total RNA was extracted from 50 mg of the bovine tissue using TRI Reagent^® ^(Molecular Research Centre, Inc. USA) according to the manufacturer's instructions. The RNA was dissolved in 20 μl of 0.1% DEPC treated water and then subjected to deoxyribonuclease I (DNase I) treatment (Qiagen, Chatsworth, CA, USA). Re-extraction of RNA was carried out using phenol-chloroform. The RNA pellet was finally dissolved in 20 μl of nuclease-free water. The quality and quantity of the total RNA was evaluated on a 1.2% agarose gel stained with ethidium bromide and on a NanoDrop^® ^ND-1000 Spectrophotometer (Thermo Fisher Scientific Inc. MA, USA), respectively. Samples with a 260/280 ratio ≥ 2.0 were used for further analysis.

### Quantitative real time RT-PCR (qRT-PCR)

Initially the cDNA was synthesized using oligo(dT)_20 _primer with 1.0 μg of total RNA in a final reaction volume of 20 μl using the Superscript™ III First-strand synthesis system for reverse transcriptase-polymerase chain reaction (RT-PCR) (Invitrogen Corp., San Diego, CA, USA). Using gene specific primers for the bovine *NPY *gene [GeneBank: AY491054] (Forward – 5' CGG CTC TCA CCC CTA GAA 3', reverse – 5' TCT GCC TGG TGA TGA GAT 3') and bovine *GAPDH *gene [42], the qRT-PCR assay was performed in an ABI 7300 Real-Time PCR system (Applied Biosystems, Foster City, CA, USA). The reaction was carried out with 0.5 μl cDNA in a 25 μl final reaction volume with 1 × Power SYBR^® ^Green master mix (Applied Biosystems) and 0.2 μM of each of the forward and reverse primers. The thermal cycle conditions were 94°C for 30 sec followed by 60°C for 1 min, for 40 cycles, followed by dissociation of the product at 96°C for 2 min. The relative quantities of the *NPY *gene transcript in different bovine tissues were normalized against the relative quantities of the *GAPDH *gene in those tissues as previously described [42].

### Full length cDNA synthesis and amplification of the 5' and 3' cDNA regions

Full length cDNA was synthesized from 2.5 μg total RNA using a GeneRacer™ Kit (Invitrogen Corp.) and following manufacturer's instructions. Briefly, the total RNA was dephosphorylated using 10 U of calf intestinal phosphatase and followed by removal of the mRNA cap structure by treating with 0.5 U tobacco acid pyrophosphatase enzyme. At the final step of RNA modification, an RNA oligonucleotide adaptor (5' CGA CUG GAG CAC GAG GAC ACU GAC AUG GAC UGA AGG AGU AGA AA 3') was ligated to the decapped mRNA using 5 U of T4 RNA ligase. Every step of RNA modification was followed by a clean-up step using phenol:chloroform. Full length cDNA was synthesized from the modified RNA using Superscript™ III First-strand synthesis system as described earlier. However, instead of the conventional oligo(dT)_20 _primer used before, GeneRacer Oligo dT primer which has an anchor sequence (5' GCT GTC AAC GAT ACG CTA CGT AAC GGC ATG ACA GTG 3') with the oligo (dT)_24 _was used.

Amplifications of the 5' end of the *NPY *gene transcript (5' RACE) were carried out using a combination of the 5' adaptor primer (5' GGA CAC TGA CAT GGA CTG AAG GAG TA 3') as a forward primer and a gene specific primer (sequence as above) as reverse primer. For the amplification of the 3' end (3' RACE), the gene specific forward primer (sequence as above) and 3' adaptor primer (5' CGC TAC GTA ACG GCA TGA CAG TG 3') were used. The PCR conditions for both 5' and 3' RACE-PCR were; 94°C for 2 min followed by forty cycles of denaturation (94°C for 30 sec), annealing (57°C for 30 sec) and amplification (70°C for 1 min 30 sec). The PCR products were assayed on a 1.2% agarose gel. The PCR product of the 5' and 3' RACE-PCR were cloned into a plasmid vector using TOPO TA cloning^® ^kit (Invitrogen Corp.) following the manufacturer's instructions. Sequencing of the plasmid DNA was carried out in both forward and reverse directions as described below.

### Identification of SNPs in the bovine *NPY *gene

#### Animals and genomic DNA preparation

The animals (total n = 40) used for identification of the SNPs in the bovine *NPY *gene comprised of unrelated pedigree bulls (n = 34) used for performance evaluation by the Irish Cattle Breeding Federation (ICBF) at the National Beef Performance Station (NBPS), Tully, Co. Kildare, Ireland and one breed of *B. indicus *(n = 6) (Table 4). Blood samples were collected from the jugular vein of the cattle in BD Vacutainers (Vacutainer Systems, UK) and stored at -20°C until used for DNA extraction. Genomic DNA was extracted from 200 μl of blood using GenElute™ Blood Genomic DNA kit (Sigma-Aldrich Corp. St. Louis, MO, USA) following manufacturer's instructions. At the final step, the genomic DNA was eluted with 200 μl nuclease free water and store at -20°C. The quality and quantity of the genomic DNA was assessed on a 0.8% agarose gel and using a NanoDrop^®^ND-1000 Spectrophotometer (Thermo Fisher Scientific Inc.), respectively.

**Table 4:** Breeds of beef cattle used for determination of the diversity of SNPs in the coding and non-coding region of bovine *NPY *gene

Species	Breed	*(n)*
		
*Bos taurus*		34
	Limousin	6
	Simmental	6
	Charolais	5
	Aberdeen Angus	4
	Hereford	3
	Parthenais	3
	Salers	3
	Aubrac	1
	Belgian Blue	1
	Blonde d'Aquitaine	1
	Shorthorn	1
*Bos indicus*	Local breeds	6
	Total	40

#### PCR amplification of the genomic region of the bovine *NPY *gene

The nucleotide sequence of the bovine *NPY *gene (including the 5'and 3' UTR) was obtained from the GeneBank through alignment of the contig [GeneBank: NC_007302.1] with the available bovine *NPY *gene sequence [GeneBank: AY491054]. Seven sets of PCR primers (Table 5) were generated using the web-based software Primer3  to amplify a total of 8361 nt genomic region. The primers were designed in such a way as to provide an overlap of ~100 nt between any two consecutive fragments. PCR amplification of the different fragments of the bovine *NPY *gene was performed in a PTC-225 DNA Engine Tetrad (MJ Research, Inc. Massachusetts, USA) with 0.75 μL of the genomic DNA template in a 50 μl final reaction volume. The PCR reaction contained 1× PCR buffer, 1.5 mM MgCl_2_, 0.2 μM dNTP mix, 0.2 μM each of the forward and reverse primers and 0.2 unit of Platinum *Taq *DNA polymerase (Invitrogen Corp.). The PCR cycle conditions were 94°C for 45 sec, 55°C for 45 sec and 72°C for 1.5 min for forty cycles followed by a final extension of 72°C for 10 min for all fragments.

**Table 5:** The sequences of the oligonuclotides and the size of the corresponding PCR products used for sequencing of different regions of the bovine *NPY *gene

Primer	Nucleotide sequence	Product size (base)
		
NP0	For 5'CCAAAACCAAGCTGGGAAAAG 3'	1427
	Rev 5'GGTCCAGTAGCAGAGCCAAG 3'	
NP1	For 5'GGGCAGACCAAAACTGAAAA 3'	1252
	Rev 5'CACCAAGTGGGCATTTTCTT 3'	
NP2	For 5'TGCCCAGACTGAAGTGAAAAG 3'	1160
	Rev 5'GGAGGGCTCCAATTTAATCAC 3'	
NP3	For 5'TCCTAGGCATTACTTTGTTCAGTC 3'	1090
	Rev 5'AGGTACCCAGAGCAGCATCA 3'	
NP4	For 5'ACCCCCAGGGTGATTCTAAC 3'	1009
	Rev 5'TGCACATTACTGCTGCTGAA 3'	
NP5	For 5'TGGACCAGTAGGTTTCCTTCA 3'	1153
	Rev 5'GCAGGCGTCTTTTCACTTTC 3'	
NP6	For 5'ACTTGGGGCTTTCTTGGAGT 3'	1020
	Rev 5'CTTCCAGCCTGTCAAACACA 3'	
NP7	For 5'CAGCAGCCTTTTCAACAGTTT 3'	1115
	Rev 5'TGCTACAGGGATTTGGTAAGC 3'	

#### Sequencing of the genomic/plasmid DNA

For sequencing of the PCR products, 40 μl of the PCR product was cleaned up using Gen Elute™ PCR clean-up kit (Sigma-Aldrich Corp.) following the manufacturer's instructions. The quality and quantity of the purified PCR product was assessed as described earlier. Using both forward and reverse primer sets, separate sequencing reactions were performed on the clean PCR product (20 ng/100 bases of DNA to be sequenced). For the plasmid DNA, 200 ng of the plasmid DNA was sequenced in both directions using plasmid-specific standard sequencing primers. Sequencing was performed by MWG-Biotech, Germany. Sequence data with PHRED quality score >20 (conform the accuracy of >99% of the base called) was used for multiple sequence comparison and SNP identification. SNPs were identified through comparison of the sequence data from all animals using Molecular Evolutionary Genetic Analysis (MEGA) v 4.0 software [43].

### Statistical Analysis

The nucleotide diversity in the promoter, exon, intron and 3' (UTR) regions of the bovine *NPY *gene was calculated considering the normalized number of variant sites as previously described [44,45]. Accordingly, the normalized number of variant sites

$$\theta =\text{K}/\sum_{\text{i}=1}^{\text{n}-1}{\text{i}}^{-1}\text{L}$$

where, 'K' is the observed number of variant sites, 'L' is the total sequence length and 'n' is the number of chromosome sequenced. Basic population statistics including the allele and genotype frequencies were determined by analysis of the genotype data using Arlequin 3.11 [46]. The haplotype analysis was performed using EM algorithm in the Arlequin software.

## Authors' contributions

Both authors actively participated in the design of the study and in the writing of the manuscript. BB performed all the experimental steps and TS supervised the SNP analysis.
